# Two Cases of Lead Colic

**Published:** 1850-03

**Authors:** R. A. Spencer

**Affiliations:** Monticello, Ind.


### ARTICLE II.

*Two Cases of Lead Colic.*—By R. A. Spencer, M.D., of Monticello, Ind.

Daniel Collier and his wife complained on the 15th of Nov. of an intermitting pain in the stomach and about the umbilicus, for which I ordered to each a cathartic of Scammony to be followed, so soon as catharsis was produced, by a full dose of Morphine.

Upon the 16th, I received notice that the medicine had not acted, and that both were worse. I suspected, as soon as I saw the family to-day, that the disease under consideration was Lead Colic, and that it had been produced upon the man by the use of xx grs. of Acet. Plumbi, given with Camphor and Opii, some days before, for profuse night-sweats; but this did not account for the woman's symptoms, and I inqured of her whether she had taken any of the medicine sent to Mr. Collier, and she answered positively, no. I had used Acet. Plumbi with Opium for many years without any bad symptoms following it, and I could not easily believe this, even in the woman's case, to be from the medicinal use of lead. Upon a faithful search, we were able to discover, what at least satisfied me, of the cause of this disease in both patients. The family used a jug glazed with the oxide of lead for the purpose of keeping their vinegar in; the children used but little of this, for they said it had not a good taste. None of them have presented the symptoms of Lead Cholic. Upon inspection, we found the glazing entirely corroded and dissolved. Be the cause of Mr. Collier's disease what it may, if this was not the cause of *Mrs. Collier's* sickness, I cannot conceive what is.

These patients, some years ago, were the subjects of salivation, and hence Mercury, could not be used to any extent, as it quickly produced constitutional symptoms. The treatment, for the first four days, consisted in fomentations to the abdomen, full doses of opium in some shape, and the occasional use of cathartics, which were made to act only in large and frequent doses. In these cases there was no blueness of the edge of the gums at any time. I determined to try the effect of Sulphuric Acid, in the gentleman's case, after every thing had failed to give permanent relief, and the pain ceased at once, the bowels moved spontaneously, notwithstanding that the whole farago of opiates were suspended from the time of the first dose of the dilute Acid. The man was soon restored to health. If this is the experience of other physicians I shall be puzzled not a little, to know how this remedy effects such signal triumphs over a disease in which even our vaunted opium stands powerless. It may scatter its roses upon the pillow of the sick, allay the pangs of the dying, and quiet the irritable nerves, while Dame Nature, and her help-mate, Man, conduct the wandering functions back to health; but it is considerably inferior to the Sul. Acid, in this abominable and harrassing disease. The great remedy of a recent writer in the American Journal, was Opium,—it has been emphatically *the remedy* of the Profession.

Mrs. Collier was still treated with opium, cathartics, &c., until the disease seemed to be incorrigible, and then was put upon the use of Alum, all other treatment suspended, except an occasional cathartic, for some days she was comfortable, and gave promise of speedy convalescence; but pains came on in the arms, at the elbow joint, running down the forearm, and Morphine was given. You may wonder why I did not give the Acid. In answer, I have only to say, I felt like making a test of the comparative value of the two remedies.

From this on, pains measurably ceased in the bowels.—They moved spontaneously. The pains in the arms were only transient, and in a few weeks ceased, without paralysis or other permanent effect. In fact, the amendment dated from the use of Alum, though I give the Acid the preference, as far as one case can be invoked for proof, but the last remedy was quite satisfactory.

Thirty or forty cases might furnish objections to these remedies, but here is a faithful account of their effects in two cases.

